# Fatty Acid Amide Hydrolase Single Nucleotide Polymorphism rs324420A/C Associations with Alzheimer’s Disease

**DOI:** 10.1002/alz.091588

**Published:** 2025-01-03

**Authors:** Daniel K Mori‐Fegan, Yuen Yan Wong, Shiropa Noor, Che‐Yuan Wu, Ruth A Ross, Walter Swardfager

**Affiliations:** ^1^ LC Campbell Cognitive Neurology Research Unit, Sunnybrook Research Institute, Toronto, ON Canada; ^2^ University of Toronto, Toronto, ON Canada; ^3^ Hurvitz Brain Sciences Program, Sunnybrook Research Institute, Toronto, ON Canada; ^4^ Sandra Black Centre for Brain Resilience and Recovery, Sunnybrook Research Institute, Toronto, ON Canada

## Abstract

**Background:**

The endocannabinoid system has demonstrated roles in Alzheimer’s Disease (AD), such as modulation of inflammation. Fatty Acid Amide Hydrolase (FAAH) is the enzyme responsible for the rapid inactivation of the endocannabinoid anandamide into arachidonic acid and ethanolamine. In doing so, FAAH modulates the concentration of anandamide and influences neurobehavioral functions and physiological conditions such as nociception and inflammatory responses. A missense single nucleotide polymorphism in the FAAH gene rs324420‐A>C (Minor allele frequency = 26.4%) is associated with higher levels of anandamide, reduced susceptibility to PTSD and increased risk of substance use disorders. However, the relationship between rs324420 and AD progression has not been explored. In AD, FAAH methylation is lower resulting in increased activity, and FAAH inhibition is suggested to drive microglial polarisation towards anti‐inflammatory phenotypes. rs324420 increases proteolytic degradation, thus reducing functionality of FAAH. Due to lowered FAAH activity, we hypothesise that rs324420 minor allele carriers will have slower neurodegeneration and slower cognitive decline in AD.

**Methods:**

This retrospective, longitudinal, observational study used data from the Alzheimer’s Disease Neuroimaging Initiative (ADNI). Patients diagnosed with mild cognitive impairment or mild AD (MCI or AD) from ADNI2/GO/3 were selected. Linear regression models evaluated baseline differences between rs324420 minor allele carriers (AC/CC) and major allele homozygotes (AA) controlling for sex, age, baseline cognitive status, APOE‐e4 status and baseline intracranial volume. Longitudinal associations were assessed as interactions between SNP and visit number (baseline to 48 months) in mixed models.

**Results:**

In Aβ‐positive patients with AD or MCI, carrying the rs324420 minor allele was associated with smaller nucleus accumbens volume (F_(1,311)_ = 15.7, p = 0.018) at baseline. Longitudinally, several brain regions showed less volumetric decline in minor allele carriers over 48 months: caudal anterior cortex (F_(1,163)_ = 5.18, p = 0.024), fusiform gyrus (F_(1,85.6)_ = 5.28, p = 0.024), rostral anterior cortex (F_(1,59.9)_ = 6.41, p = 0.014) and nucleus accumbens (F_(1,84.7)_ = 4.75, p = 0.032). Carriers of the minor allele also showed less decline MMSE score over 48 months (F_(1,544.15)_ = 5.13, p = 0.024).

**Conclusions:**

Slower rates of cognitive and neuroanatomical decline in amyloid positive patients with MCI or mild dementia due to AD suggest a potential protective role of the rs324420 minor allele in the FAAH gene. Further investigation is warranted, including cognitive, anatomical, fluid biomarker and neuropsychiatric symptom profiles.

